# Electroacupuncture promotes skin wound repair by improving lipid metabolism and inhibiting ferroptosis

**DOI:** 10.1111/jcmm.17811

**Published:** 2023-06-12

**Authors:** Weibin Du, Zhenwei Wang, Yi Dong, Huahui Hu, Huateng Zhou, Xiaofen He, Jintao Hu, Yong Li

**Affiliations:** ^1^ Research Institute of Orthopaedics the Affiliated Jiangnan Hospital of Zhejiang Chinese Medical University Zhejiang China; ^2^ Hangzhou Xiaoshan Hospital of Traditional Chinese Medicine Zhejiang China; ^3^ Shaoxing TCM Hospital Affiliated to Zhejiang Chinese Medical University Zhejiang China; ^4^ Key Laboratory of Acupuncture and Neurology of Zhejiang Province, Department of Neurobiology and Acupuncture Research The Third Clinical Medical College, Zhejiang Chinese Medical University Zhejiang China; ^5^ Orthopaedics and Traumatology Department Hangzhou TCM Hospital Affiliated to Zhejiang Chinese Medical University Zhejiang China

**Keywords:** electroacupuncture, ferroptosis, lipid metabolism, oxidative stress, wound repair

## Abstract

Lipid metabolism plays an important role in the repair of skin wounds. Studies have shown that acupuncture is very effective in skin wound repair. However, there is little knowledge about the mechanism of electroacupuncture. Thirty‐six SD rats were divided into three groups: sham‐operated group, model group and electroacupuncture group, with 12 rats in each group. After the intervention, local skin tissues were collected for lipid metabolomics analysis, wound perfusion and ferroptosis‐related indexes were detected and finally the effect of electroacupuncture on skin wound repair was comprehensively evaluated by combining wound healing rate and histology. Lipid metabolomics analysis revealed 37 differential metabolites shared by the three groups, mainly phospholipids, lysophospholipids, glycerides, acylcarnitine, sphingolipids and fatty acids, and they could be back‐regulated after electroacupuncture. The recovery of blood perfusion and wound healing was faster in the electroacupuncture group than in the model group (*p* < 0.05). The levels of GPX4, FTH1, SOD and GSH‐PX, which are related to ferroptosis, were higher in the electroacupuncture group than in the model group (*p* < 0.05). The levels of ACSL4 and MDA were lower in the electroacupuncture group than in the model group (*p* < 0.05). Electroacupuncture may promote skin wound repair by improving lipid metabolism and inhibiting ferroptosis in local tissues.

## 1 INTRODUCTION

The skin is the largest organ of the body and is the first line of defence against external invasion and has properties that protect against microbial, mechanical, chemical, osmotic and thermal damage.^1^ The integrity of the skin may be disrupted by trauma, lacerations, cuts, or contusions, resulting in skin wounds. When the skin is damaged to the dermis, the process of wound healing results in the formation of scars that are distinguishable from the surrounding skin affecting the aesthetics and may produce various symptoms such as inflammation, erythema, pruritus and pain, which adversely affect the quality of life of patients and cause high global health system costs.^2^, ^3^ Despite the advances in wound healing, the complexity of this process remains a significant clinical problematic obstacle. Some common treatments used to promote wound healing include surgery, corticosteroid injections, radiation therapy, and local silicone and interferon injections.^4^ These therapies help to improve symptoms but have some disadvantages such as side effects that are difficult for patients to accept. Therefore, there is a need to find a safe and effective complementary and alternative therapy to promote the healing of skin wounds.

Acupuncture is an important part of Chinese traditional medicine, and has achieved more significant clinical efficacy in a variety of clinical diseases. Electroacupuncture is an important innovation and advancement of Chinese traditional medicine, which is based on acupuncture plus electrical stimulation, with the dual benefits of acupuncture and electrical stimulation. Electrical stimulation is a green therapy that mobilizes endogenous currents in the skin, corrects the internal environment of wounds, and accelerates wound repair, which can reduce infection, improve cellular immunity, increase perfusion and accelerate wound healing.^5^ Acupuncture can promote wound healing through anti‐inflammatory effects and increase re‐epithelialization and angiogenesis.^6^ Clinical studies have demonstrated that the use of microcurrent electrical stimulation therapy in patients with difficult‐to‐heal wounds accelerates wound healing and reduces pain.^7^ However, the role and mechanism of electroacupuncture for cutaneous wound healing are less reported and need further study.

The lipid‐based skin barrier plays a crucial role in human life. These barrier lipids fill the uppermost skin stratum corneum and consist mainly of ceramides, free fatty acids and cholesterol.^8^ This multilayered, tightly packed mixture of lipids forms an effective barrier to the environment, thus protecting the body from harmful external factors such as pathogens, allergens, chemical compounds and UV radiation and preventing internal water loss.^9^MS lipidomics, which detects the mass‐to‐charge ratio of a large number of lipids, has become the tool of choice to address the complexity of the skin lipidome. Chromatography‐mass spectrometry (LC–MS) has significantly increased the capability of analytical platforms, thus providing a sensitive and specific tool for identifying and quantifying skin lipids.^10^, ^11^ To investigate the overall role of electroacupuncture in skin wound repair, we applied LC–MS to analyse the dynamic expression of lipid metabolites in rats with total skin defects and explored the important target lipid metabolites and associated lipid pathways of electroacupuncture for skin wound repair, as well as the mechanism of ferroptosis response.

## 2 METHODS

### 2.1 Experimental animals

Thirty‐six male SD rats (weight 160 ± 20 g) were grouped in the Animal Experimentation Centre of Zhejiang University of Traditional Chinese Medicine for 1 week after acclimatization. SD rats were purchased and fed, and other animal procedures followed the animal research guidelines of the National Institutes of Health and the Animal Research Committee and approved by the Experimental Animal Ethics Committee of Zhejiang University of Traditional Chinese Medicine (NO.IACUC‐20220221‐19).

### 2.2 Model preparation

Combined with the group's previous modelling basis, a full‐layer skin defect model was prepared. After anaesthesia, the modelling area (2 cm on the left and right side of the spine) was fixed at five points, trimmed and dehaired, rinsed with saline, disinfected with iodophor and deiodinated with ethanol. A 1*1 cm square model of the full skin defect was created using surgical scissors. Postoperatively, the wound was naturally hemostatic, and the wound was kept dry to prevent wound infection.

### 2.3 Grouping and processing

Thirty‐six SD rats were randomly divided into three groups of 12 rats each according to the table of random number methods, as follows: sham‐operated group, model group and electroacupuncture group. (1) Sham‐operated group: only hair clipping was done in the modelling area, and no wound model was made. (2) Model group: full skin defect model was prepared and only iodophor was given daily for routine disinfection to prevent wound infection. (3) Electroacupuncture group: Based on the operation of the model group, electroacupuncture treatment was started on the same day. The central point of the wound and the normal skin at the edge of the wound (the edge of the midpoint of the four sides of the square wound) were selected and given electroacupuncture stimulation. The negative electrode was located at the centre and the positive electrode was located at the edge of the midpoint of the four sides of the square wound. Continuous pulses with a frequency of 2 Hz and an output current of 0.3 mA were used to stimulate each point in turn once a day for 20 min to prevent wound infection.

### 2.4 LC–MS model preparation

Skin tissue was taken from the moulded area after 7 days in each group. After 30 min of ultrasonication in an ice bath, the samples were vortexed for 1 min and placed in a refrigerator at −20°C overnight. The next day, the centrifuge was pre‐cooled to 4°C and centrifuged at 13000 rpm for 15 min. 300 μL of skin tissue supernatant was taken, blown to dryness with nitrogen, and 150 μL of isopropanol was re‐dissolved and centrifuged at 13000 rpm for 15 min. 100 μL of supernatant was taken into the injection vial for sample injection. The supernatant of the homogenate was centrifuged in equal amounts, blown dry with nitrogen, and re‐dissolved to prepare QC samples. The liquid conditions were set and the samples were analysed by UHPLC‐QTOF/MS.

### 2.5 UHPLC‐QTOF/MS analysis

#### 2.5.1 Chromatography

Chromatographic separation was performed on an ExionLC system (AB Sciex). A Waters Acquity HSS T3 column (2.1 × 100 mm, 1.7 μm) was applied at the temperature of 55°C. Mobile phase A: water‐acetonitrile (40:60, containing 0.1% formic acid with 5 mM ammonium formate); mobile phase B: isopropanol‐acetonitrile (90:10, containing 0.1% formic acid with 5 mM ammonium formate) The gradient was optimized as follows: 0–13 min from 30% to 80% B, 13–20 min from 80% to 90% B, 20–21 min from 90% to 100% B, 21–26 min at 100% B, then back to the initial ratio of 30% B and maintained with additional 8 min for re‐equilibration. The injection volume of all samples was 2 μL.

#### 2.5.2 Mass spectrometry

To provide high‐resolution detection, a X500B Q‐TOF mass spectrometer (AB Sciex) equipped with an electrospray ionization source (Turbo Ionspray) was applied. MS detection was implemented both in negative and positive ion mode with the mass rang at m/z 150–1050. The parameters of the mass spectrometer were summarized as follows: gas1 and gas2, 45 psi; curtain gas, 35 psi. Heat block temperature, 550°C; ion spray voltage, −4.5 kV in negative mode and 5.5 kV in positive; declustering potential, 50 V; collision energy, ± 35 V; and the collision energy spread (CES) was ± 15 V. To monitor the reproducibility and stability of the acquisition system, QC samples were prepared by pooling small aliquots of each sample. The QC specimens were analysed every six samples throughout the whole analysis procedure.

#### 2.5.3 Data processing

The raw profiles were extracted by SCIEX OS Analytics and transformed into a data matrix, mainly including information on the mass‐to‐charge ratio (m/z) and retention time (Rt) and peak area (intensity). All data were normalized by the total peak area and the excel sheet was generated for subsequent metabolome analysis. To reduce signal interference from chance errors, variables with RSD ≥40% in QC were excluded in excel first.

The excel files were imported into SIMCA 14.1 (Umetrics, Umeå, Sweden) software for multivariate mathematical and statistical analysis. Principal component analysis (PCA) was used to observe the overall distribution of the samples. In addition, the consistency of the samples within the group was analysed by PCA‐Class analysis. Generally, when a sample falls outside the ‘2‐std. dev.’ line under a principal component, the sample was considered abnormal data, and the sample data was considered to be excluded before the subsequent analysis.

The OPLS‐DA replacement test statistically analyses the validity of the OPLS‐DA model when Q2 intersects with the y‐axis at a negative value, indicating that the model is valid, which in turn screens for differential metabolites. Based on this model, the differential variables were screened according to the variable projection importance index (VIP value), and the variables with VIP >1 were generally considered to be meaningful variables causing the differences. The variables with a high impact on OPLS‐DA model building were further screened by the partial correlation coefficient (pcorr). Finally, the screened variables were tested for significance (Mann–Whitney Test), and a *p*‐value <0.05 was considered a significant difference variable. potential markers were identified by HMDB (http://www.hmdb.ca/) and LIPID MAPS (https: //www.lipidmaps.org/). All differential metabolites involved in the three groups were summarized by Venn diagrams. Heat maps of the differential metabolites were produced to visualize the high and low levels of the response of the compounds after the intervention, and corresponding bar charts were produced for further analysis. Based on the results of the significantly different metabolites screened and identified, the compound name results for each group were imported into Metabo Analyst 5.0 (http://www.MetaboAnalyst.ca/) for metabolic pathway analysis.

### 2.6 Laser doppler perfusion imaging test

The groups were examined at 1, 4, 7 and 14days using laser Doppler perfusion imaging with a distance of 10 cm between the probe and the test object and an imaging range of 1.0 cm × 1.0 cm. and the PIMSoft software was applied to the record, analyse, process, and store the body surface flow maps. The changes in perfusion in each group were compared.

### 2.7 Postoperative wound observation

The model and electroacupuncture groups were photographed at 4, 7 and 14 days using a digital camera, and trauma healing was analysed using image‐pro Plus 6.0 image analysis software. Percentage of trauma healing = [(original trauma area—trauma area at the time of observation) ÷ original trauma area] × 100%.

### 2.8 Histological testing

Skin tissues of each group were taken from the moulded area after 7 days, fixed with 4% PFA solution for more than 24 h, dehydrated, and paraffin‐embedded to make 4um sections. HE and Masson staining were performed, and after completing the steps, the sections were dehydrated and sealed, observed under the microscope and photographed for comparison.

### 2.9 Immunohistochemical examination immunohistochemical staining

Skin tissues of the modelled area were taken from each group after 7 days, and the fixation, embedding and sectioning methods were the same as the histological testing steps. After paraffin depletion and rehydration, the antigen was heat‐retrieval with 0.01 M citrate buffer for 15 min. 5% BSA was used to closure, and primary antibodies were incubated with GPX4, FTH1 and ACSL4 (1:200 each) overnight at 4°C, after washing with PBS for three times, incubated for 2 h at room temperature with secondary antibodies, and washing with PBS for three times again. The secondary antibody was further visualized with DAB buffer for 20 min. After sealing, observe under the microscope and take photos for comparison.

### 2.10 Western blot analysis

The skin tissues of each group were taken from the moulded area after 7 days, and the protein expression of GPX4, FTH1 and ACSL4 in each group was analysed by extracting total tissue protein; measuring protein concentration by BCA method; SDS‐PAGE electrophoresis; membrane transfer; immuno‐reaction (incubation of primary and secondary antibodies); chemical reflection (colour development by two mixed reagents of ECLA and ECLB); taking pictures on the machine; and analysing by AI image software, etc. The comparison was performed.

### 2.11 Detection of biochemical indic

The skin tissues of each group were taken from the moulded area after 7 days, mixed 1:9 according to the volume of skin tissues and 0.9% saline, and mechanically homogenized under ice water bath conditions to prepare a 10% homogenate, centrifuged at 2500 ~ 3000 rpm for 10 minutes, and the supernatant was taken and quantified according to the instructions of the MDA, SOD and GSH‐PX kits.

### 2.12 Statistical Analysis

All of the experimental results were expressed as the mean ± SD (standard deviation). All statistical analyses were performed using SPSS 21.0 software. The significance of differences between groups was determined by a 2‐tailed unpaired Student's t‐test or repeated measures analysis of variance when samples were not distributed normally. A value of *p* < 0.05 was considered to be statistically significant.

## 3 RESULTS

### 3.1 Repeatability and Stability of the UHPLC‐QTOF/MS Method

**FIGURE 1:**
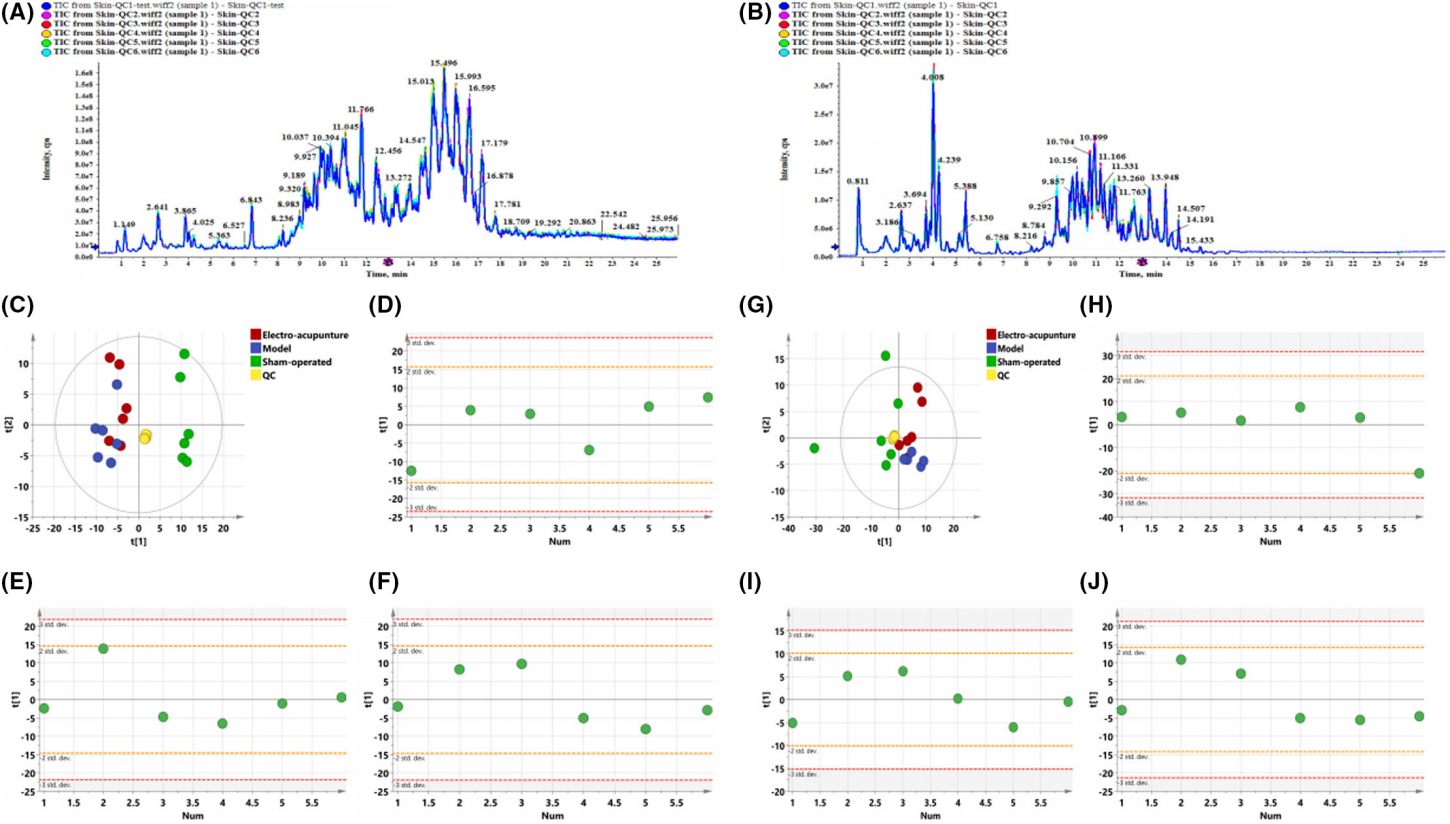
(A,B). The BPC diagram of QC samples in positive and negative ion mode. (A) Positive ion mode. (B) Negative ion mode. Figure 1(C–F). PCA analysis of negative ions. (C) PCA analysis of all samples (R^2^X 0.671, Q^2^ 0.439). (D) PCA‐class analysis of Sham‐operated group. (E) PCA‐class analysis of Model group, (F) PCA‐class analysis of Electro‐acupuncture group. Figure 1(G–J). PCA analysis of positive ions. (G) PCA analysis of all samples (R^2^X 0.858, Q^2^ 0.529). (H) PCA‐class analysis of Sham‐operated group. (I) PCA‐class analysis of Model group. (J) PCA‐class analysis of Electro‐acupuncture group.

Figure 1(A),1(B) show the QC Base Peak Chromatograms (BPC) in positive and negative ion modes. The Base Peak Chromatogram is a continuous depiction of the most intense ion intensity obtained at each time point, and the BPC contains the ion intensity as well as the retention time of the ion in the chromatogram. Figure 1(C),Figure 1(G) show the PCA plots of all samples in positive and negative ion mode, respectively. It can be seen that the QC samples are more tightly clustered, indicating that this experiment has good stability and reproducibility. The consistency of the samples within the group was analysed by PCA‐Class analysis, and all data could be retained as seen from the results in Figure 1(C–F) and Figure 1(G–J).

### 3.2 Results of differential metabolite analysis

#### 3.2.1 Different metabolite profiles in different groups

**FIGURE 2:**
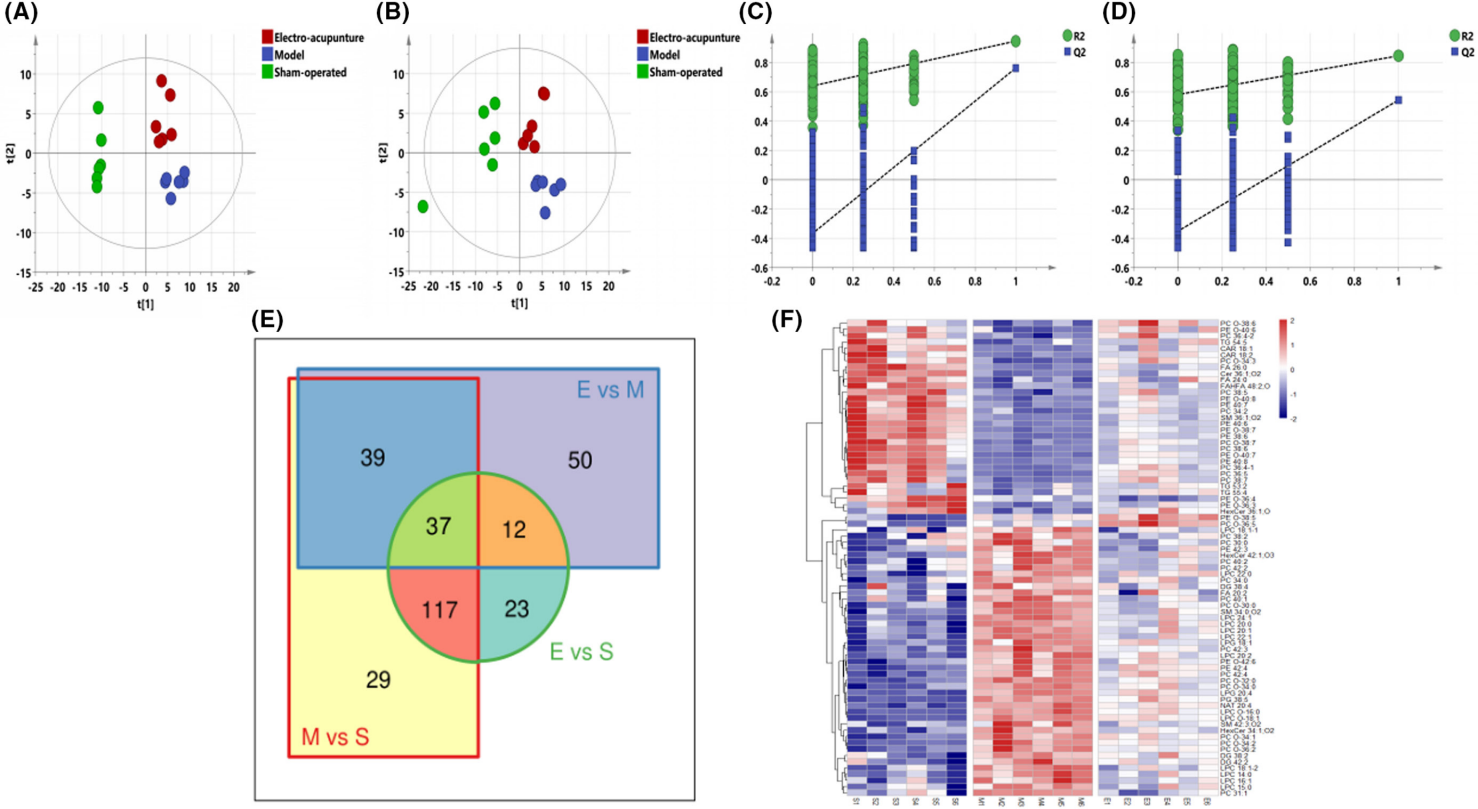
(A–D). PLS‐DA diagram of all samples. (A) PLS‐DA plot in negative ion mode (R2X 0.721, R2Y 0.963, Q2 0.844), (B) PLS‐DA plot in positive ion mode (R2X 0.777, R2Y 0.907, Q2 0.741), (C) Negative ion replacement test, (D) Positive ion replacement test. (E) Venn diagram of three groups of differential metabolites. The red box indicates Model versus Sham‐operated. The blue box indicates Electro‐acupuncture versus Model, and the green circle indicates Electro‐acupuncture versus Sham‐operated. (F) Differential lipid heat map. A red colour indicates a higher response, and a blue colour indicates a lower response.

**FIGURE 3:**
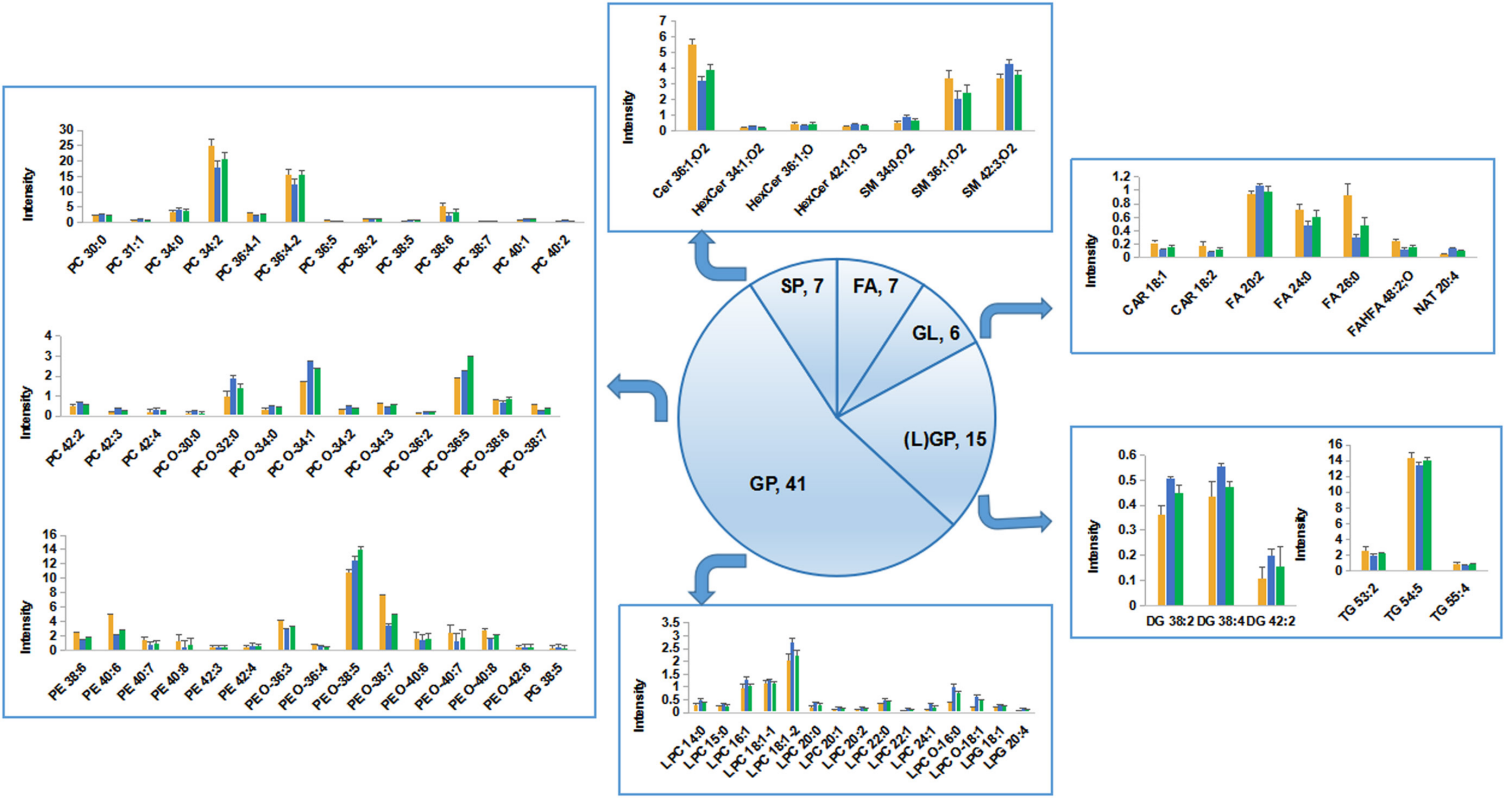
Comparison of differential metabolite response.

PLS‐DA analysis was performed for all groups to observe the trend of the overall metabolic group of the three groups. The results are shown in Figure 2(A–D), Model, Electro‐acupuncture, and Sham‐operated were distinguishable between all three groups, and the sham‐operated group was relatively more distant, suggesting migration of the lipid group after modelling. Lipids also changed after electroacupuncture treatment, and a tendency to move closer to the sham‐operated group could be observed from the distribution of positive ion PLS‐DA, suggesting that there might be some lipid retracing. All differential metabolites involved in the three groups were summarized by Venn Diagram (Figure 2E): the red box surrounded by the number of differential metabolites for Model versus Sham‐operated, 222 in total, the blue box surrounded by the number of differential metabolites for Electro‐acupuncture versus Model, 138 in total, and the green circle surrounded by the number of differential metabolites for Electro‐acupuncture versus Sham‐operated was 189. There are 37 differential metabolites common to all three groups, and 76 metabolites that overlap between Model versus Sham‐operated (red) and Electro‐acupuncture versus Model (blue). Of these 76 differential lipids, 73 were reversed after electroacupuncture treatment, and the results are shown in Table 1. As seen in the heat map (2F): the difference between the sham‐operated and model groups was obvious, with more elevated lipids than decreased lipids, and most lipids were back‐regulated to some extent after electroacupuncture, but the difference was significantly weaker than Model versus Sham‐operated. As seen in the histogram (Figure 3), differential phospholipids (GP) were the most abundant, but there was no consistent increase or decrease, showing an overall metabolic disorder; lysophospholipids (LGP) and diglycerides (DG) were elevated in the model group as a whole and retracted after electroacupuncture, while acylcarnitine (CAR) and triglycerides (TG) were decreased in the model group and retracted after electroacupuncture; the rest, such as sphingolipids (SP) and fatty acids (FA), showed metabolic disorders but could be partially retracted after electroacupuncture.

**TABLE 1:** 76 coincident differential metabolite information.

NO.	Name	Class	Fold(M/S)	Fold(E/M)	Reversed
1	CAR 18:1	FA	0.54	1.35	Y
2	CAR 18:2	FA	0.46	1.63	Y
3	FA 20:2	FA	1.13	0.92	Y
4	FA 24:0	FA	0.67	1.25	Y
5	FA 26:0	FA	0.33	1.57	Y
6	FAHFA 48:2;O	FA	0.52	1.29	Y
7	NAT 20:4	FA	2.34	0.79	Y
8	DG 38:2	GL	1.40	0.89	Y
9	DG 38:4	GL	1.28	0.85	Y
10	DG 42:2	GL	1.81	0.78	Y
11	TG 53:2	GL	0.76	1.13	Y
12	TG 54:5	GL	0.94	1.04	Y
13	TG 55:4	GL	0.80	1.15	Y
14	LPC 14:0	GP	1.55	0.80	Y
15	LPC 15:0	GP	1.43	0.82	Y
16	LPC 16:1	GP	1.37	0.80	Y
17	LPC 18:1–1	GP	1.12	0.90	Y
18	LPC 18:1–2	GP	1.36	0.82	Y
19	LPC 20:0	GP	1.81	0.80	Y
20	LPC 20:1	GP	2.06	0.74	Y
21	LPC 20:2	GP	1.93	0.78	Y
22	LPC 22:0	GP	1.51	0.85	Y
23	LPC 22:1	GP	2.26	0.68	Y
24	LPC 24:1	GP	2.99	0.65	Y
25	LPC O‐16:0	GP	2.50	0.77	Y
26	LPC O‐18:1	GP	3.29	0.76	Y
27	LPG 18:1	GP	1.37	0.88	Y
28	LPG 20:4	GP	2.84	0.76	Y
29	PC 30:0	GP	1.17	0.85	Y
30	PC 31:1	GP	1.32	0.76	Y
31	PC 34:0	GP	1.21	0.91	Y
32	PC 34:2	GP	0.72	1.15	Y
33	PC 36:4–1	GP	0.71	1.24	Y
34	PC 36:4–2	GP	0.80	1.23	Y
35	PC 36:5	GP	0.46	1.69	Y
36	PC 38:2	GP	1.15	0.89	Y
37	PC 38:5	GP	0.76	1.15	Y
38	PC 38:6	GP	0.41	1.46	Y
39	PC 38:7	GP	0.54	1.53	Y
40	PC 40:1	GP	1.33	0.80	Y
41	PC 40:2	GP	1.35	0.80	Y
42	PC 42:2	GP	1.28	0.83	Y
43	PC 42:3	GP	2.02	0.72	Y
44	PC 42:4	GP	1.77	0.87	Y
45	PC O‐30:0	GP	1.72	0.72	Y
46	PC O‐32:0	GP	1.89	0.75	Y
47	PC O‐34:0	GP	1.61	0.82	Y
48	PC O‐34:1	GP	1.62	0.86	Y
49	PC O‐34:2	GP	1.59	0.82	Y
50	PC O‐34:3	GP	0.69	1.33	Y
51	PC O‐36:2	GP	1.90	0.77	Y
52	PC O‐36:5	GP	1.21	1.30	FALSE
53	PC O‐38:6	GP	0.82	1.31	Y
54	PC O‐38:7	GP	0.42	1.64	Y
55	PE 38:6	GP	0.54	1.28	Y
56	PE 40:6	GP	0.42	1.32	Y
57	PE 40:7	GP	0.53	1.25	Y
58	PE 40:8	GP	0.37	1.62	Y
59	PE 42:3	GP	1.25	0.84	Y
60	PE 42:4	GP	1.70	0.86	Y
61	PE O‐36:3	GP	0.73	1.12	Y
62	PE O‐36:4	GP	0.71	0.84	FALSE
63	PE O‐38:5	GP	1.16	1.11	FALSE
64	PE O‐38:7	GP	0.45	1.42	Y
65	PE O‐40:6	GP	0.83	1.19	Y
66	PE O‐40:7	GP	0.51	1.43	Y
67	PE O‐40:8	GP	0.57	1.31	Y
68	PE O‐42:6	GP	1.33	0.89	Y
69	PG 38:5	GP	2.66	0.80	Y
70	Cer 36:1;O2	SP	0.57	1.23	Y
71	HexCer 34:1;O2	SP	1.73	0.73	Y
72	HexCer 36:1;O	SP	0.68	1.32	Y
73	HexCer 42:1;O3	SP	1.92	0.70	Y
74	SM 34:0;O2	SP	1.64	0.79	Y
75	SM 36:1;O2	SP	0.60	1.19	Y
76	SM 42:3;O2	SP	1.28	0.84	Y

Abbreviations: CAR, acyl carnitines, DG, di(acyl|alkyl)glycerols; FA: fatty acids, FAHFA, branched fatty acid esters of hydroxy fatty acids, NAT, N‐acyl taurines; TG, Tri(acyl|alkyl)glycerols, LPC, lyso glycerophosphocholines, LPG, lyso glycerophosphoglycerols, PC, glycerophosphocholines, PE, glycerophosphoethanolamines, PG, glycerophosphoglycerols, Cer, ceramides, HexCer, hexosyl ceramides, SM, sphingomyelins.

### 3.3 Metabolomics pathway analysis of SD rat skin tissues

**FIGURE 4:**
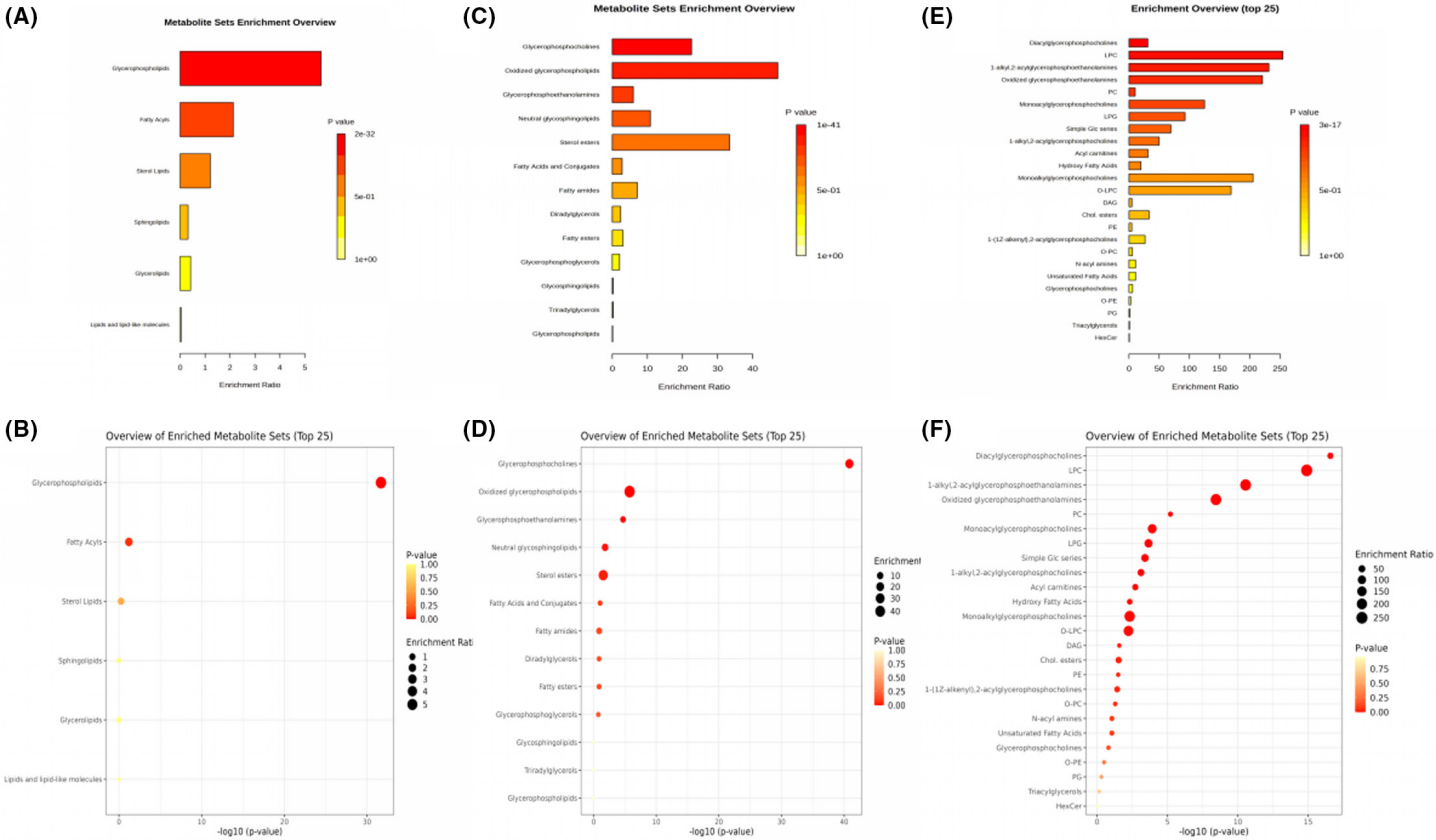
Lipid metabolism enrichment analysis. (A) Top lipid classification, enrichment analysis bar chart. (B) Top lipid classification, enrichment analysis dot plot. (C) Intermediate lipid classification, enrichment analysis bar chart. (D) Intermediate lipid classification, enrichment analysis dot plot. (E) The lowest lipid classification, enrichment analysis bar chart. (F) The lowest lipid classification, enrichment analysis dot plot.

The results of pathway enrichment analysis from different lipid classification levels showed that from the highest level of lipid pathway analysis (Figure 4A,B), electroacupuncture mainly back‐regulated the lipids associated with six pathways: phospholipids, fatty acyl, sterolipids, sphingolipids, glycerides and lipid‐like substances (the redder the colour, the more significant the expression of the pathway; the longer the bar or the larger the point, the more lipids matched in the pathway). And the metabolic pathways were further refined and analysed from the intermediate and lowermost lipid categories, see Figure 4(C–F).

### 3.4 Postoperative wound perfusion volume and area changes

**FIGURE 5:**
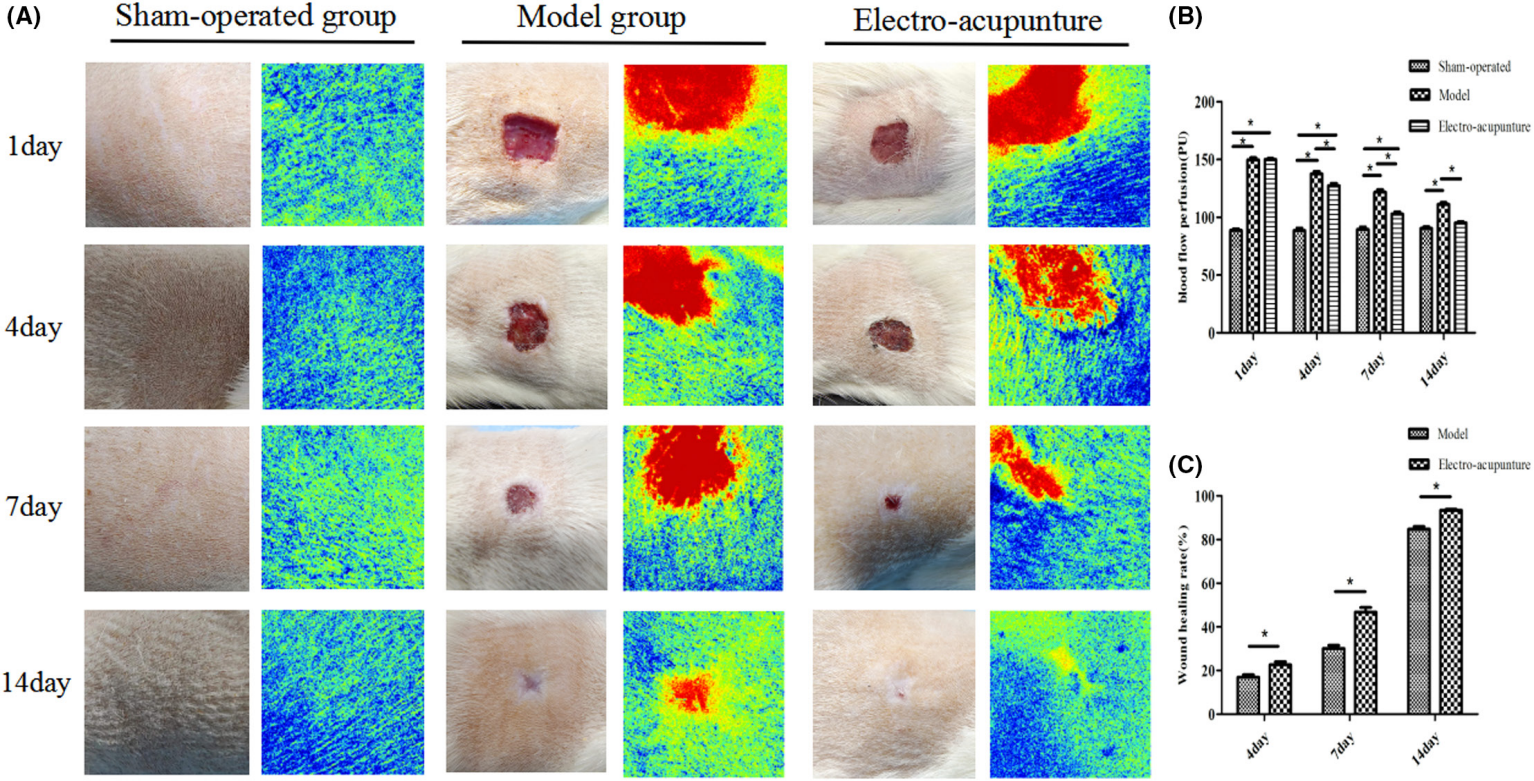
(A) Wound conditions and corresponding blood perfusion images of the three groups at different time points after surgery. (B) Comparison of wound blood perfusion volume in the three groups at different time points after surgery. (C) Comparison of wound healing rate between Model group and Electro‐acupunture at different time points after surgery.

On postoperative day 1, the haemoperfusion volume in the model and electroacupuncture groups was comparable but significantly greater than that in the sham‐operated group, and the difference was statistically significant (*p* < 0.05). On postoperative days 4 and 7, the perfusion volume of the model group and electroacupuncture group gradually decreased, but the perfusion volume of the model group was greater than that of the other two groups, and the difference was statistically significant (*p* < 0.05). On the 14th postoperative day, the perfusion volume of the model group and electroacupuncture group further decreased, and the electroacupuncture group was close to the sham‐operated group, and the difference was not statistically significant (*p*>0.05), while the perfusion volume of the model group was still greater than the other two groups, and the difference was statistically significant (*p*<0.05), and the results are shown in Figure 5(A,B). On postoperative days 4, 7 and 14, the wound healing rate of the electroacupuncture group was faster than that of the model group, and the difference was statistically significant (*p* < 0.05), and when the postoperative day 14 was reached, the wound in the electroacupuncture group was close to complete healing, and the results are shown in Figure 5C.

### 3.5 Histological and immunohistochemical results

**FIGURE 6:**
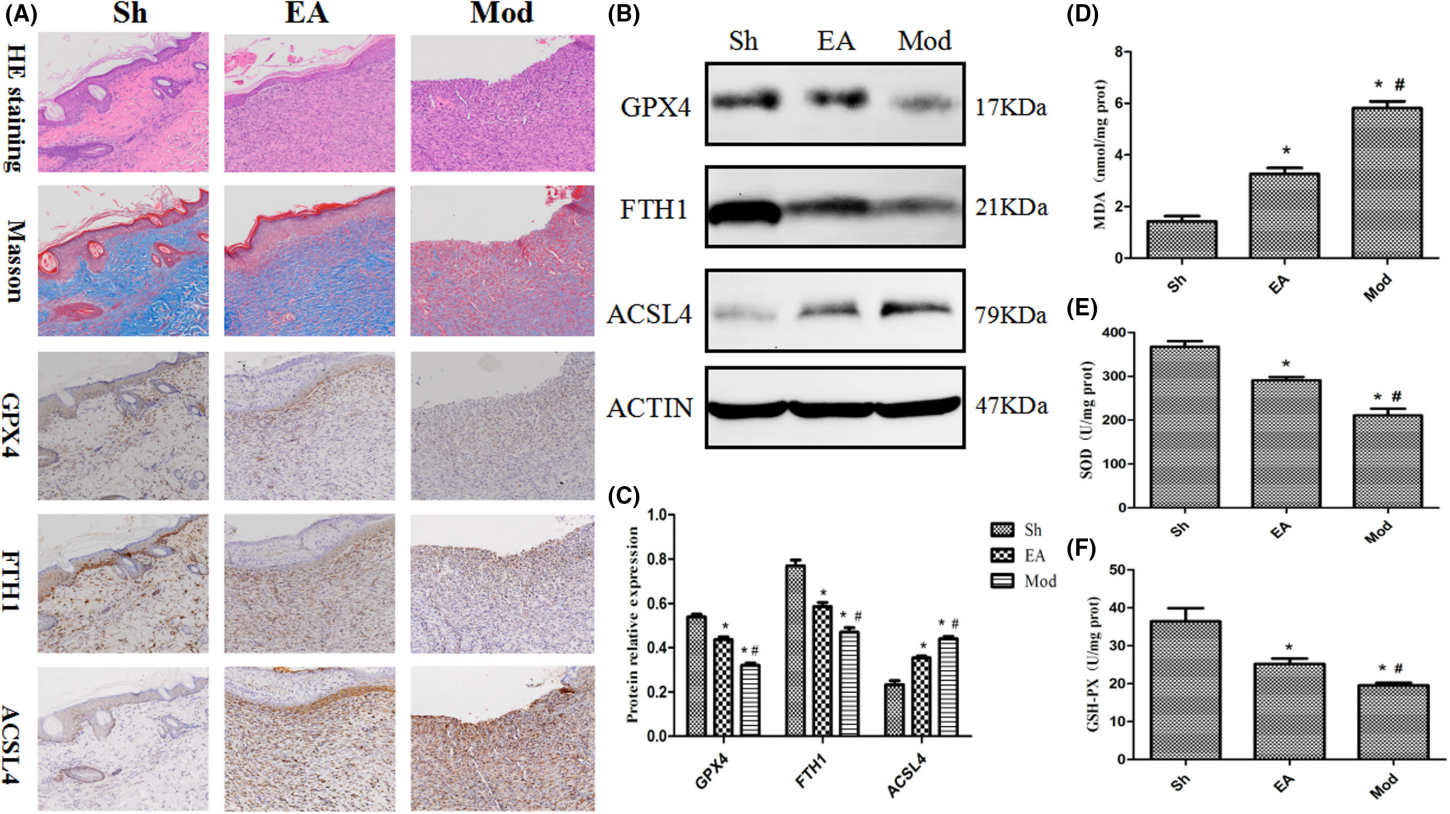
(A) Histological and immunohistochemical results of each group (X200). (B) Western blot was used to detect related proteins in each group and their grey values were compared. (C) Comparison of detection results of biochemical indexes in each group.

On the 7th postoperative day, epithelial cells and fibroblasts were hyperplastic in the electro‐acupuncture group, collagen fibres were densely arranged, muscle fibres were more abundant, and a thinner new epidermal layer was visible. And the skin repair status of the model group was weaker than that of the electroacupuncture group. The expression of GPX4 and FTH1 was the highest in the sham‐operated group and gradually decreased in the electro‐acupuncture group and the model group. the expression of ACSL4 was the lowest in the sham‐operated group and gradually increased in the electro‐acupuncture group and the model group. See Figure 6A.

### 3.6 Western blot related protein and biochemical index results

**FIGURE 7:**
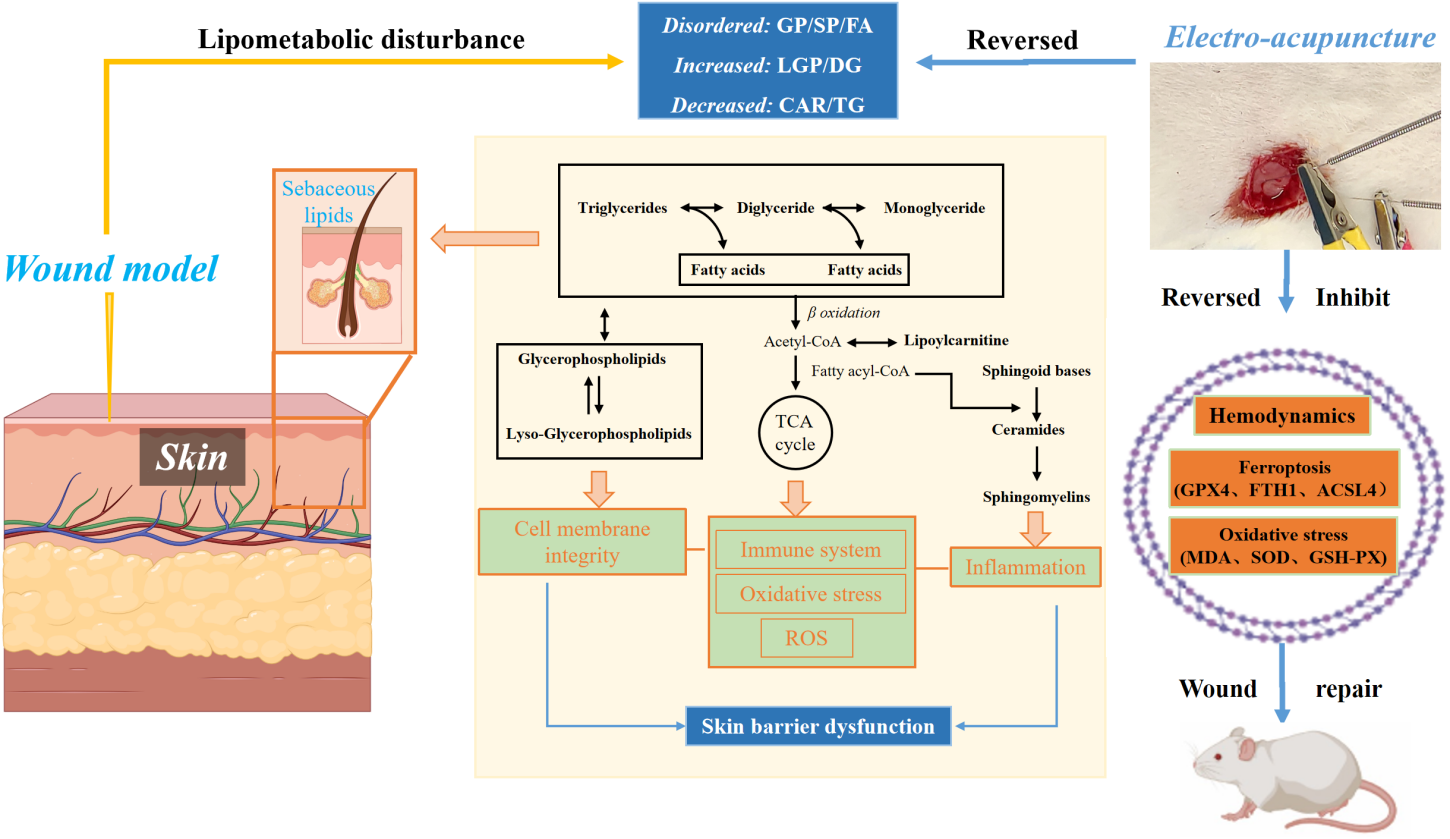
Schematic diagram of electroacupuncture promotes skin wound repair by improving lipid metabolism and inhibiting ferroptosis.

Consistent with the immunohistochemical results, the protein expression of GPX4 and FTH1 was highest in the sham‐operated group and gradually decreased in the electro‐acupuncture and model groups on postoperative day 7 (*p* < 0.05). the protein expression of ACSL4 was lowest in the sham‐operated group and gradually increased in the electro‐acupuncture and model groups (*p* < 0.05), see Figure 6(B‐C). The levels of MDA were lowest in the sham‐operated group and gradually increased in the electro‐acupuncture and model groups (*p* < 0.05), and the levels of SOD and GSH‐PX were highest in the sham‐operated group and gradually decreased in the electroacupuncture and model groups (*p* < 0.05), see Figure 6(D–F). Based on the analysis of lipid metabolic pathways and ferroptosis results, a schematic diagram (Figure 7) was drawn in combination with the results of KEGG and literature review to investigate the potential mechanism of action of electroacupuncture on skin wound repair.

## 4 DISCUSSION

The physiological functions of various parts of the skin are inextricably linked to the physiological structure and function of the basic skin compounds, including proteins, lipids, carbohydrates and nucleic acids. These molecules have a variety of functions, such as enzymatic activity and signal Lipids are highly diverse biomolecules that play a crucial role in the formation and function of cell membranes, metabolism and cell signalling.^12^ Lipids likewise play an irreplaceable role in maintaining the skin barrier, supporting skin structure, defending against external attacks and pathogenic pathogens and regulating skin inflammation.^9^ It has been found that electroacupuncture can stimulate fibroblast proliferation, recombine collagen fibres, promote angiogenesis and regulate the changes in gene expression profile during wound healing, thus accelerating wound repair.^13^, ^14^ In the present study, we demonstrated for the first time that electroacupuncture can target lipid metabolism, particularly phospholipid metabolism, to promote the healing of total skin defects in rats.

In this study, lipid metabolism disorders were found in both electro‐acupuncture and model groups. 222 differential metabolites were found in Model versus Sham‐operated, 138 differential metabolites in Electro‐acupuncture versus Model, 138 differential metabolites in Electro‐acupuncture versus Sham‐operated 189 metabolites. There were 37 differential metabolites common to all three groups, and 76 metabolites that overlapped between Model versus Sham‐operated and Electro‐acupuncture versus Model. Seventy‐three of these 76 differential lipids were reversed after electroacupuncture treatment, suggesting a pullback effect of electroacupuncture on skin tissue metabolism. These metabolites that were back‐regulated after electroacupuncture were mainly phospholipids, lysophospholipids, glycerides, acylcarnitine, sphingolipids and fatty acids. Further metabolic pathway analysis revealed that electroacupuncture mainly back‐regulated lipids related to six pathways: phospholipids, fatty acyl, sterol lipids, sphingolipids, glycerol esters and lipid‐like substances, which was consistent with the results of differential metabolite analysis. In addition to this, the electroacupuncture group recovered faster than the model group in terms of blood perfusion and wound healing. The levels of GPX4, FTH1, SOD and GSH‐PX, which are related to ferroptosis, were higher in the electroacupuncture group than in the model group (all *p* < 0.05). The levels of ACSL4 and MDA were lower in the electroacupuncture group than in the model group (all *p* < 0.05). It is suggested that electroacupuncture inhibited ferroptosis and promoted skin wound healing. Therefore, our study may provide a safer and more effective complementary and alternative therapy for skin wound healing and provide an experimental basis for the study of the mechanism of electroacupuncture to promote skin wound healing.

Phospholipids are the main components of biological membranes and are widely found in all cell membranes in the human body, playing an essential role in the normal metabolism and life activities of cells. Phospholipids are amphiphilic lipids with polar head groups derived from phosphoric acid and also have long hydrocarbon chains, which make the molecule hydrophobic. It not only serves as a major structural component of cell membranes but also regulates various biological processes such as cell proliferation, apoptosis, immunity, angiogenesis and inflammation responsible for cell signalling.^15^, ^16^ Phospholipids are hydrolyzed into lysophospholipids (Lyso‐PLs) and fatty acids by phospholipase.^17^ Reactive oxygen species (ROS) generated by ultraviolet (UV) radiation can lead to skin barrier dysfunction, while sphingolipid‐containing lacto‐phospholipids can reduce skin barrier damage by regulating heme oxygenase‐1 (HO‐1) and reducing ROS levels through milk phospholipid‐mediated activation of nuclear factor erythroid‐2‐related factor 2 (Nrf2).^18^ Our study found that phospholipids, especially lysophospholipid metabolites and metabolic pathways, were back‐regulated by electroacupuncture intervention. It is suggested that electroacupuncture may promote the balance of phospholipid metabolism (Phospholipid deposition and excessive hydrolysis of phospholipids) by inhibiting excessive oxidative stress, restoring the integrity of skin cell membranes, and inhibiting skin inflammatory processes. This may be a mechanism of metabolic action of electroacupuncture to promote wound repair.

Fatty acids are oxidized through β‐oxidation to produce acetyl CoA, which is completely oxidized by the tricarboxylic acid cycle to produce CO2 and H2O and release energy. Fatty acid oxidation is divided into three stages, activation‐transfer‐oxidation and fatty acids need to be catalysed by lipid acyl CoA synthase to generate lipid acyl CoA first, then transported into mitochondria with the help of carnitine, and complete the transformation of lipid acyl CoA‐lipid acyl carnitine (CAR)‐lipid acyl CoA under the action of carnitine lipid acyltransferase, and enter the matrix across the inner mitochondrial membrane to participate in oxidation^19^, ^20^The decrease in fatty acids and complex phospholipids may be due to a dramatic decrease in 3‐carbon intermediates (glycerol‐based) in the glycolytic pathway, which generally provides the backbone for the synthesis of these lipids.^21^ Palmitic acid, lauric acid and oleic acid in free fatty acids increase the release of antimicrobial peptides in the sebaceous glands, thus achieving an antimicrobial effect and preventing the entry of external harmful substances into the organism.^22^ Sciadonic acid (SA), an anti‐inflammatory fatty acid analogue of arachidonic acid, has also been shown to have anti‐inflammatory effects in human skin, accelerating stimulus‐induced healing and improving skin barrier function under controlled clinical conditions.^23^, ^24^ Triglyceride and fatty acids are important components of sebaceous lipids (Sebaceous lipids), accounting for 57% of their composition, freshly secreted sebum does not contain fatty acids, but is produced by the hydrolysis of triglyceride by bacteria and yeast on the surface of the skin after sebum secretion.^25^ Sebum has a role in moisturizing as well as maintaining and restoring the skin barrier, and in addition triglycerides in skin lipids can inhibit the expression of matrix metalloproteinase‐1 and inflammation‐associated protein cyclooxygenase‐2 (COX‐2) and interleukin‐1b (IL‐1b) after UV irradiation, suggesting that triglyceride play an important role in skin repair.^26^ The hydrolysis of triglyceride produces one molecule of fatty acids with one molecule of diacylglycerol, which has also been reported in the literature to be associated with the formation of the stratum corneum.^27^ Dysfunctional mitochondria and aberrant energy metabolism can also lead to oxidative stress and ROS accumulation, which can lead to inflammation.^28^, ^29^, ^30^ A growing number of studies have shown that mitochondrial dysfunction and oxidative stress are key features of aging in various tissues, including the skin.^31^, ^32^ Our study found that fatty acid‐like and triglycerides metabolites and metabolic pathways were back‐regulated after electroacupuncture intervention. Since both fatty acid‐like and triglycerides metabolism are important pathways of the tricarboxylic acid cycle, it suggests that electroacupuncture may promote the tricarboxylic acid cycle, facilitate the restoration of mitochondrial function, inhibit the occurrence of oxidative stress, facilitate the restoration of the skin barrier, and improve the immune function of the body, thus accelerating the healing of skin wounds. This may be another mechanism of metabolic action of electroacupuncture to promote skin wound healing.

Ferroptosis is a novel form of programmed cell death different from apoptosis, necrosis and autophagy, first proposed by Stockwell's team in 2012^33^ and officially defined by the cell death nomenclature committee in 2018. The main mechanism of ferroptosis is excessive intracellular iron accumulation causing lipid peroxidation, which leads to abnormal mitochondrial structure and causes cellular dysfunction.^34^ ferroptosis is characterized by the accumulation of intracellular iron overload and iron‐dependent lipid peroxidation. In addition, ferroptosis leads to the inhibition of oxidoreductases, particularly glutathione peroxidase 4 (GPX4),^35^ a lipid peroxide scavenger that protects cell membranes from peroxidative damage by accelerating the reduction of lipid peroxides using glutathione as a cofactor.^36^ Li et al.^37^ have found that electroacupuncture can inhibit ferroptosis by regulating oxidative stress (MDA, SOD, GSH, GPX4) and iron‐related proteins (Tf, TfR1, FTH1), so as to improve ischemic brain injury. Thus, ferroptosis may adversely affect skin wound healing by modulating the action of ROS and lipid peroxidation products. In our study, we showed that all ferroptosis‐related indices were back‐regulated after electroacupuncture intervention, suggesting that electroacupuncture may promote wound healing and restore the skin barrier by inhibiting oxidative stress and suppressing ferroptosis. This corroborates with the lipid metabolism results.

Our study results suggest that metabolites and metabolic pathways such as phospholipids, lysophospholipids, glycerides, acylcarnitines, sphingolipids and fatty acids can be back‐regulated after electroacupuncture intervention and may play a key role in the mechanism of action of electroacupuncture to promote skin wound repair. The electroacupuncture intervention facilitated the restoration of blood perfusion during rat wound repair, promoted the proliferation of rat epithelial cells and fibroblasts, attenuated lipid peroxidation and inflammatory activity in rat trabecular skin granulation tissue, improved local tissue lipid metabolism and inhibited ferroptosis. Although there are some limitations, for example, more experimental studies are urgently needed to verify the mechanisms by which electroacupuncture interventions regulate lipid metabolism and iron death in the skin, and no clinical trials have been conducted to verify efficacy. However, our results provide new insights into the treatment of skin wound repair, and also provide experimental basis for subsequent clinical trials.

## AUTHOR CONTRIBUTIONS

**weibin du:** Funding acquisition (lead); project administration (lead); writing – original draft (lead); writing – review and editing (lead). **Zhenwei Wang:** Data curation (equal); validation (equal); writing – original draft (equal). **Yi Dong:** Conceptualization (equal); methodology (equal); writing – original draft (equal). **Huahui Hu:** Data curation (equal); methodology (equal). **Huateng Zhou:** Resources (equal); validation (equal). **Xiaofen He:** Investigation (equal); software (equal); supervision (equal). **Jintao Hu:** Conceptualization (equal); data curation (equal); formal analysis (equal). **Yong Li:** Project administration (lead); resources (lead); writing – review and editing (lead).

## FUNDING INFORMATION

This work is supported by National Natural Science Foundation of China (NO. 81904053). Special Research Project of the Affiliated Hospital of Zhejiang Chinese Medical University (NO. 2021FSYYZY43). Hangzhou Medical and Health Technology Planning Project (NO. B20220021, B20200032, A20220507), Hangzhou Science and Technology Planning Project (NO. 2020ZDSJ0042, 20220919Y084). Zhejiang Province Traditional Chinese Medicine Science and Technology Project (NO. 2023ZR046, 2022ZB232), Research Project of Zhejiang Chinese Medical University (NO. 2021JKZKTS057B).

## CONFLICT OF INTEREST STATEMENT

All the authors declare that they have no conflicts of interest.

## Data Availability

All data generated and/or analyzed during this study are included in this published article.
